# Antiparkinsonian effects of the "Radiprodil and Tozadenant" combination in MPTP-treated marmosets

**DOI:** 10.1371/journal.pone.0182887

**Published:** 2017-08-30

**Authors:** Anne Michel, Jean-Marie Nicolas, Sarah Rose, Michael Jackson, Peter Colman, Willy Briône, David Sciberras, Pierandrea Muglia, Dieter K. Scheller, Martin Citron, Patrick Downey

**Affiliations:** 1 UCB BioPharma, Braine L’Alleud, Belgium; 2 King’s College, Institute of Pharmaceutical Science, London, United Kingdom; 3 UCB BioPharma, Slough, United Kingdom; Centre national de la recherche scientifique, FRANCE

## Abstract

**Objective:**

Investigate a combination of two clinically tested drugs, the NR2B antagonist Radiprodil and the A_2A_ antagonist Tozadenant in the MPTP-treated marmoset model of Parkinson’s Disease (PD).

**Background:**

In PD, there remains a need for the development of non-dopaminergic drugs to effectively treat the motor symptoms without the induction of L-Dopa-induced motor complications.

**Methods:**

Clinically relevant doses of Radiprodil and Tozadenant were given both alone and in combination without the addition of L-Dopa, and the antiparkinsonian efficacy of the treatments was assessed in a primate model of PD.

**Results:**

When compared to the drugs tested alone, the drug combination led to a significant increase of motor activity and an improvement of motor disability in MPTP-treated marmosets. In addition, the motor restoration brought about by the combination was almost completely devoid of dyskinesia. Interestingly, treated primates were not overstimulated, but were able to move normally when motivated by the exploration of novel objects.

**Conclusion:**

We have demonstrated in a primate model that, the “Radiprodil/Tozadenant” combination significantly improves motor activity, extending previous results obtained in unilaterally lesioned 6-OHDA-rats. The strength of the preclinical data accumulated so far suggests that the use of such an A_2A_ and NR2B antagonist combination could bring significant motor improvement to PD patients, without inducing the motor complications induced by L-Dopa therapy. Although encouraging, these preclinical data need to be confirmed in the clinic.

## Introduction

L-Dopa given together with a peripheral dopa-decarboxylase inhibitor still remains the gold standard treatment for the motor symptoms of Parkinson’s disease (PD). However, long term treatment with this combination invariably leads to debilitating side effects related to motor complications (i.e. on-off motor fluctuations and dyskinesia) [1]. Long term experience with L-Dopa demonstrates that the majority of treated patients experience dyskinesia, a percentage that can rise to 80 to 90% after 10 years of treatment [2]. Consequently, the indentification of efficacious non-dopaminergic pharmacotherapies which avoid these severe and predictable motor complications remains a significant unmet need in the treatment of PD patients. For this purpose, one could envisage the use of drugs which don’t directly stimulate the up-regulated dopaminergic receptors in the lesioned striatum.

Over the last fifteen years, the adenosine A2 (A_2A_) receptor has emerged as an attractive target for PD treatment, given its functional interaction with dopamine receptors in the basal ganglia [3,4]. In preclinical studies, A_2A_ receptor antagonists, administered without L-Dopa, have shown potential antiparkinsonian activity in rodent [5–7] and primate [8,9] models of PD. Similarly, NR2B, a specific subunit of the N-methyl-D-Aspartate (NMDA) receptor, has also been identified as an important player in PD symptomatology [10,11]. NR2B antagonists, have been shown to have antiparkinsonian efficacy against motor symptoms in both rats [12] and primates when used in the absence of L-Dopa [13]. As there is evidence suggesting that the NMDA and A_2A_ receptors interact, at least within the striatum [14], the therapeutic potential of the combined administration of A_2A_ and NR2B antagonists was assessed in the unilateral 6-OHDA-lesioned rat PD model [15]. These rat data demonstrated that, when given in the absence of L-Dopa, an NR2B and an A_2A_ antagonist combination treatment was not only able to substantially restore the quantity of movement but could also significantly improve the quality of the movement when compared to L-Dopa. Furthermore, unlike L-Dopa, the combination treatment did not induce any involuntary movements in rats [16].

Unfortunately, some antiparkinsonian effects observed in preclinical models were not reproduced in the clinic. For example, A_2A_ antagonists failed to demonstrate significant effects when given as monotherapy to patients [17] and while NR2B antagonists were shown to be active against L-Dopa-induced dyskinesia (LIDs) [18], the only compound reportedly tested in patients in the absence of L-Dopa (MK-0657) failed to show significant antiparkinsonian efficacy [19]. These clinical outcomes may suggest that monotherapy with either an A_2A_ or an NR2B antagonist would not be sufficient to restore motor activity on their own.

In order to evaluate the effect of the A_2A_/NR2B combination in a model which more closely resembles the clinical situation, the current study evaluated the antiparkinsonian effects of the combined administration of Tozadenant and Radiprodil, given in the absence of L-Dopa, in the 1-methyl-4-phenyl-1, 2, 3, 6,-tetrahydropyridine (MPTP)-treated marmoset model of PD. Tozadenant is a selective A_2A_ receptor antagonist which recently entered a Phase 3 trial [20]. It has previously been shown to provide statistically significant clinical benefit on “on-time” and to improve the UPDRS part III score in PD patients [21]. In contrast, Radiprodil is a NR2B-selective NMDA receptor antagonist, initially developed for the treatment of neuropathic pain. However, its clinical development for pain was stopped in phase 2 due to a lack of efficacy [22].

We hypothesized that the combination of the two above mentioned Phase 2/3 compounds, tested in a primate model, would be more efficacious against the parkinsonian symptoms than the compounds alone while being devoid of LID.

## Materials and methods

### Drugs

The vehicle was composed of 0.1% (w/v) Tween 80 (Sigma-Aldrich, UK), 0.1% (w/v) silicone antifoam 1510 US (Dow Corning, US), 20% (w/v) Kleptose HPB (Roquette, Belgium), 1.0% (w/v) methylcellulose (Sigma-Aldrich, UK) in water. Tozadenant (Biotie, US) was administered as a suspension at a dose volume of 2 mL/kg by oral gavage. Radiprodil (UCB, Belgium) was administered as a solution at a dose volume of 2 mL/kg by oral gavage. Dosing suspensions were prepared approximately 18 hrs in advance and homogenised by using a polytron and magnetic stirrer. L-DOPA methyl ester hydrochloride (4.0 mg/kg, Sigma-UK lot number SLBD2579V, dose calculated as free base) was dissolved in a 10% sucrose solution containing benserazide HCl (5mg/ml, see above) and administered in a volume of 2mL/kg by oral gavage and prepared on the day of the experiment. Benserazide HCl (10 mg/kg, 5mg/mL, Sigma-UK lot no. BCBB8323) was dissolved in a 10% (w/v) sucrose (lot no. SZB90120 Sigma-Aldrich, UK) solution, freshly prepared on the day of the experiment.

### Animals

All experiments were performed according to the Animals Scientific Procedures Act 1986 with the guidelines of the European Community Council directive 2010/63/EU under Project Licence No 70/7146 and, were approved for this specific study by the Animal Welfare & Ethical Review Board of King’s College London. Primates were housed at King’s College London, Guy’s campus (UK) with a cage size of 163 cm in height, 140 cm of width and 90 cm in depth. The cages could be subdivided into four equal sections. Half cages (Height 81 cm) were used for severely disabled animals immediately after MPTP treatment. Following recovery from MPTP the majority of animals were housed in full height cages. Several methods were used to minimize any suffering for the primates (S1 File). At the end of the experimental period all animals were returned to the colony, and rested for at least two weeks prior to veterinary approval for continued use in further studies.

Adult common marmosets (*Callithrix jacchus*; n = 12; body weight 350-500g, Harlan UK), previously treated with MPTP (2.0mg/kg, sc) for five consecutive days and exhibiting stable motor deficits at the start of experimentation were used [23]. Animals had been previously primed to express mild to severe dyskinesia by chronic L-DOPA treatment and were not naïve to test drugs [24]. Animals were housed according to UK Home Office Guidelines in male (vasectomised) & female pairs where this was appropriate. The holding rooms were set for a 12 hours light/dark cycle (light on at 6:30 am, light off at 6:30 pm with periods of increasing/decreasing light intensity to represent dawn and dusk) with a temperature range of 25 ± 1°C and 50% relative humidity. All animals had *ad libitum* access to food pellets (*Marzuri* primate diet, Special Dietary Services) and water. They also received mashed *Marzuri* pellets and forage mix in the morning and mixed fresh fruits in the afternoon. On the days of behavioural assessment animals had free access to water but received food only at the end of the test period.

### Behavioural assessment

On the day of behavioural testing, animals were removed from their home cages between 07.15–07.30h and placed individually into the behavioural test cages and allowed to acclimatise for 1 hour for the assessment of basal activity. Following the initial drug administration behavioural assessments were then performed for 10 hours. To ensure maintenance of therapeutic plasma levels throughout the whole observation period, a second dosing was given 5 hours after the initial treatment, at which time a novel object (table tennis ball/ cotton reel) was placed into the test cage. Experiments were performed twice weekly with an intervening period of at least 2 days (wash out period). All the drug-treatment conditions were represented at every testing session. Locomotor activity, Motor disability and Dyskinesia were evaluated as described in the Supporting Information section (S1 File, “Behavioral analysis for the MPTP-treated marmosets”) and raw data are available in S2–S4 Files.

### Experimental design

Before initiating the Radiprodil & Tozadenant combination experiment, 14 primates were challenged with a dose of 8 mg/kg (po) of L-Dopa and behaviour was recorded for 5 hours. This “Pre-L-Dopa-test” was used to select the 12 subjects required for the study. Their response to the L-Dopa challenge was subsequently used as a comparator to the ensuing response to the different drug treatments and combinations. The effects of the drugs alone or in combination were assessed after acute oral gavage.

This experiment investigated the effects on motor deficits of Radiprodil plus Tozadenant twice daily at an interval of 5 hrs according to a modified Latin square design in n = 12 MPTP-treated marmosets (S1 Table). At the time of the second drugs administration a novel object (cotton reel, table tennis ball) was placed into the cage. The four different treatments were (1) Tozadenant (150 mg/kg) plus Radiprodil (2.0 mg/kg), (2) Tozadenant (150 mg/kg) plus vehicle, (3) Radiprodil (2.0 mg/kg) plus vehicle, (4) vehicle.

### Statistical analysis

The data were recorded over a period of 10 hours subdivided within two periods: pre-object placement (period 1) and post-object placement (period 2). The total scores of the activity counts of the first and second period were used as dependent variables for locomotor activity. For the disability score, to allow parametric statistical analyses, the sum of the number of observation sequences with a score below 8 (on-time activity) was taken as the dependent variable for period 1 and period 2. For locomotor activity and disability score, the effect of Radiprodil and Tozadenant were assessed with three-way mixed design ANOVA, incorporating the effect of Tozadenant (2 levels, vehicle and the dose of 150 mg/kg) and the effect of Radiprodil (2 levels, vehicle and the dose of 2 mg/kg) as between-group factors, and the Period (2 levels, pre-object and post-object) as within-subjects factors. The reliability of the between-mean differences for each relevant comparison was assessed via planned orthogonal contrasts using an F statistic. As necessary, logarithmic transformations to normalise raw data prior to the statistical analysis, were performed to more precisely meet the assumption of homogeneity of variances (Levene’s test). For the sake of clarity, however, means of the raw values are presented in the figures. Statistical significance was set at p<0.05. The dyskinesia total score was analysed with non-parametric statistics. Statistical analyses were performed using the Prism (GraphPad software) and Statistica (StatSoft Inc., OK, USA) software.

### Pharmacokinetics

At the indicated times, blood samples were collected from the femoral vein. These samples were collected in EDTA tubes, centrifuged to obtain plasma and stored at -70°C until mass spectrometry analysis (for detailed description of the methods see S1 File).

## Results

### Dose regimen selection for MPTP-treated marmosets

The doses used for the A_2A_ and NR2B antagonists combination were selected to provide the same plasma exposure values as those found to be active in the previously reported 6-OHDA rat study [15]. In the rodent PD model, the orally active dosing regimen of 3 mg/kg of Radiprodil combined with 30 mg/kg of Tozadenant was associated with plasma levels of 522 ng/mL Radiprodil and 6054 ng/mL Tozadenant (measured at 60 min). Similar exposure was achieved in the present MPTP-treated marmoset model after oral dosing of 2 mg/kg Radiprodil and 150 mg/kg Tozadenant (S1 Fig). As observed in rat, Radiprodil and Tozadenant do not show a pharmacokinetic interaction when co-administered (S2 Fig). The plasma levels measured in the marmoset were sustained (t_1/2_ ≥ 3h) and in the same range as those achieved in the Phase 2 trials with 120 mg BID Tozadenant [25] and 45 mg TID Radiprodil (unpublished; data on UCB files).

### The “Radiprodil and Tozadenant” combination improves the parkinsonian symptoms in MPTP-treated marmosets

#### Locomotor activity

**Fig 1:**
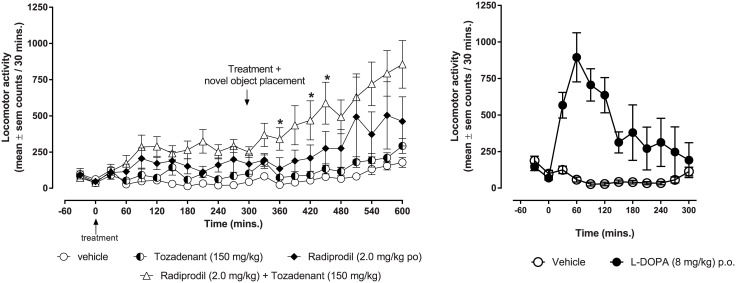
Comparison of the effects of the “Radiprodil and Tozadenant” combination with the effects of L-Dopa on the level of locomotor activity in MPTP-treated marmosets. *Left graph*: Lomotor activity (mean value ± SEM) measured over 10 hours following Radiprodil (2 mg/kg) and Tozadenant (150 mg/kg) treatment given alone or in combination in MPTP-treated marmosets (n = 12). Five hours after the first drug administration, a second administration was given and, at the same time, novel objects were introduced in the testing cage. * *versus* Radiprodil group (one-way ANOVA, p<0.01, followed by planned contrasts). *Right graph*: Locomotor activity measured over 5 hours follwing L-Dopa (8 mg/kg). p<0.001 L-Dopa *v*s Vehicle (Student test on total score).

When treated with the Radiprodil/Tozadenant combination (2 mg/kg Radiprodil and 150 mg/kg Tozadenant), MPTP-treated marmosets (n = 12), showed a significant increase in motor activity (Fig 1 left graph) in comparison to the effect of the drugs alone. In comparison with L-Dopa (Fig 1, right graph), the Radiprodil and Tozadenant combination showed a very different profile of efficacy with a slower initiation of the activity. However, this effect lasted longer and reached a similar magnitude as that observed with L-Dopa.

**Fig 2:**
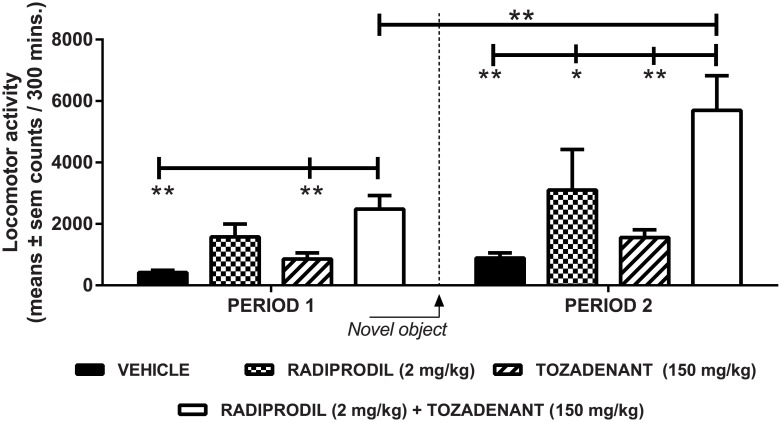
Statistical analysis of the “Radiprodil and Tozadenant” combination in MPTP-treated marmosets for the level of locomotor activity. Increased level of locomotor activity with Radiprodil (2 mg/kg) and Tozadenant (150 mg/kg) treatment when given alone or in combination in MPTP-treated marmosets (n = 12/group). Period 1 corresponds to the effect observed when the animals were in their usual testing environment. Period 2 corresponds to the period of time wherein the primates received the second drug administration and when the novel objects were put in the testing cage (Two-way mixed ANOVA followed by planned contrasts with *, p<0.05 and **, p<0.01).

Both Radiprodil and Tozadenant significantly contributed to the increased locomotor activity in MPTP-treated marmosets (significant effect of Tozadenant [F(1,44) = 5.47, p<0.05]; significant effect of Radiprodil [F(1,44) = 15.82, p<0.001]; no Tozadenant x Radiprodil interaction). The addition of a novel object to the testing environment and the second drug challenge also contributed significantly to the increase of the activity [Period: F(1,44) = 59.49, p<0.001]. Planned contrasts (Fig 2) showed that for the first period, animals treated with the Radiprodil/Tozadenant combination had a significantly higher level of locomotor activity than the vehicle and the Tozadenant group (p<0.01). By contrast, for the second period, the group treated with the Radiprodil/Tozadenant combination had significantly higher levels of locomotor activity than the groups treated with Vehicle (p<0.01), Tozadenant (p<0.01) and Radiprodil (p<0.05). Also, the Radiprodil/Tozadenant-treated group showed a significantly higher level of locomotor activity during the second period, with the object present in the test cage, in comparison to the activity measured during the first period in the absence of the object (p<0.01).

#### Motor disability

**Fig 3:**
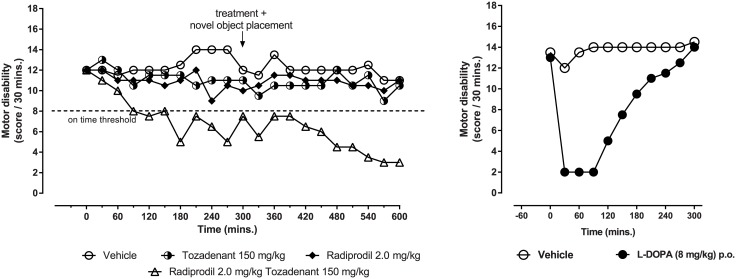
Comparison of the effects of the “Radiprodil and Tozadenant” combination with the effects of L-Dopa on the disability score in MPTP-treated marmosets. *Left graph*: Motor disability score (median value) recorded over 10 hours following Radiprodil (2 mg/kg) and Tozadenant (150 mg/kg) treatment given alone or in combination in MPTP-treated marmosets (n = 12). Five hours after the first drug administration, a second administration was given and, at the same time, novel objects were introduced in the testing cage. The score of 8 relates to the threshold below which the behaviours recorded corresponded to the definition of “on-time” activity (One-way ANOVA, p<0.01, Kruskal-Wallis). *Right graph*: Disability scored measured over 5 hours following L-Dopa (8 mg/kg). p<0.001 L-Dopa *v*s Vehicle (Mann-Whitney test on total score).

When administered together to MPTP-treated primates, both Radiprodil and Tozadenant significantly contributed to the improvement of the disability score (Fig 3, left graph). As observed previously with locomotor activity, the Radiprodil & Tozadenant combination showed a very different profile to that observed with L-Dopa (Fig 3, right graph). A delay of the improvement of motor disability was observed but, the efficacy of the drug combination lasted longer than that observed with L-Dopa. However, the drug combination did not succeed to reach a comparable amplitude of the motor improvement to that observed with L-Dopa.

**Fig 4:**
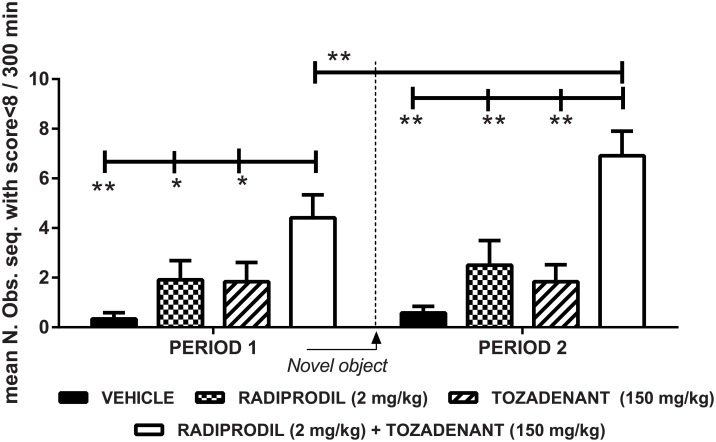
Statistical analysis of the “Radiprodil and Tozandenant” combination in MPTP-treated marmosets for the disability score. Sum of the number of observation sequences (means ± SEM) wherein the primates’ behaviour was assessed as ‘on-time’ activity (motor disability scoring<8). The behaviour was scored over 10 hours after Radiprodil (2 mg/kg) and Tozadenant (150 mg/kg) treatment given alone or in combination in MPTP-treated marmosets (n = 12/group). Period 1 corresponds to the effect observed when the animals are in the usual testing cage. Period 2 indicates the effect associated with the second drug administration and the introduction of a novel object in the testing environment (Two-way mixed ANOVA followed by planned contrasts with *, p<0.05 and **, p<0.01).

As observed in Fig 4, a significant effect of Radiprodil [F(1,44) = 13.42, p<0.001] and of Tozadenant [F(1,44) = 10.87, p<0.01] was observed but no “Radiprodil x Tozadenant” (p = 0.29) interaction. The introduction of the novel object also contributed to the significant improvement of motor disability scores [Period: F(1,44) = 5.88, p<0.05]. Planned contrasts for period 1 and 2 showed that, during these two periods, the Radiprodil/Tozadenant group displayed longer time with improved motor disability score than the vehicle, Tozadenant and Radiprodil groups (p<0.05 and p<0.01). Also, the Radiprodil/Tozadenant group showed improved motor activity at the second period in comparison to the first one (p<0.01). Consequently, these data demonstrate that the on-time duration is longer under the combination rather than under the drugs alone and the observation is reinforced when novel objects are introduced in the test cage.

#### Dyskinesia

**Fig 5:**
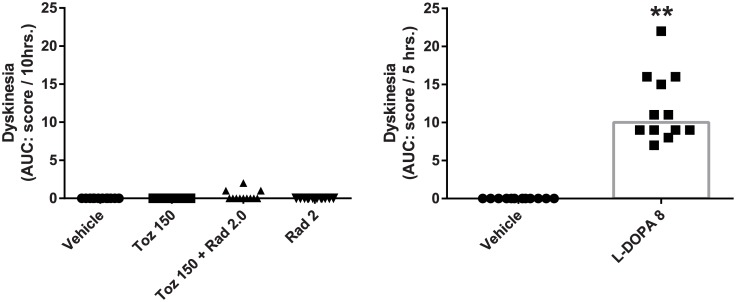
Comparison of the effects of the “Radiprodil and Tozadenant” combination with L-Dopa on the level of dyskinesia in MPTP-treated marmosets. Data are individual scores. Left graph: dyskinesia score in MPTP-treated marmosets having received Radiprodil (2 mg/kg) and Tozadenant (150 mg/kg) treatment alone or in combination over the 10-hour of behavioural recording. Right graph: dyskinesia scores recorded over 5 hours after L-Dopa treatment (8 mg/kg; **, p<0.05, Mann-Withney test).

When administered to MPTP-treated primates, both Radiprodil and Tozadenant administered alone or together did not induce a significant level of dyskinesia over the 10 hours of behavioural observation (Fig 5, left graph). Only three subjects demonstrated very minor dyskinesia. By contrast, the level of dyskinesia observed with L-Dopa was, as expected, much higher than the vehicle treatment (p<0.01) (Fig 5, right graph).

## Discussion

In the current study, we used a bilateral primate model of PD to demonstrate that the combined administration of A_2A_ and NR2B receptor antagonists significantly increases the level of motor activity and substantially improves the parkinsonian disability score. Drugs were administered orally in combination at clinically relevant doses to MPTP-treated marmosets without L-Dopa. This work confirms and extends previous studies in unilaterally-lesioned rats where the NR2B/A_2A_ antagonist combination proved to be significantly more effective than the individual drugs alone [15,16]. The most striking observation in the present primate study was the long-lasting restoration of movement which was not accompanied by any dyskinesia even though the primates had been L-Dopa primed prior to drug administration.

Radiprodil by itself had some motor effects in marmosets since it was associated with a small increase in locomotor activity, although it had no effect on motor disability scores. This observation is consistent with previous reports showing the anti-parkinsonian potential of NR2B antagonist drugs in the same animal model [12,13]. Tozadenant, by contrast, did not show any significant antiparkinsonian activity when given alone. This differs from previous studies with two other A_2A_ antagonists, Preladenant and Istradefylline, which were reported to elicit mild antiparkinsonian effects when given to primates as monotherapy [8,9,26,27].

In order to be able to demonstrate the long lasting beneficial effect of the combination on primate behaviour, the protocol of the present study was adapted to overcome the fact that the animals were habituated to their environment. The two drugs were devoid of any direct dopaminergic stimulation and thus were not expected to produce behavioural hyperstimulation. In previous experiments, performed with unilateral 6-ODHA-lesioned rats, the NR2B/A_2A_ combination significantly increased the level of locomotor activity. Instead of the typical contralateral rotations observed with dopaminergic drugs, the combination produced an increased exploratory activity accompanied by a restoration of fluid, coordinated and symmetrical quadripedal movements which looked similar to that observed with mildly stimulated non-lesioned rats [15,16,28]. In addition, we also noticed that after chronic treatment with the combination, the unilateral-lesioned rats did not develop any abnormal movements but rather developed an habituation to the testing environment resulting in decreased exploratory behaviour, making behavioural scoring difficult. However, the increase in motor activity became strongly evident when the rats were placed into a novel testing context, wherein normal exploratory activity returned, indicating that the pseudo tolerance effect resulted from the elementary learning process of *Habituation* rather than from a pharmacological effect (unpublished data). Habituation is an adaptive mechanism which requires maturation and integrity of the central nervous system [29,30]. Consequently, it can be hypothesized that the combination treatment did not disrupt the normal cognitive functioning of the lesioned rats.

As MPTP-treated marmosets were highly habituated to their testing environment, a novel object was introduced to the testing cage to encourage them to move while exploring and thus facilitate behavioural scoring. The results demonstrated that the MPTP-treated marmosets were not hyperstimulated as observed with dopaminergic drugs. They reacted to the presence of the novel object in the cage and explored it. They all showed a significant increase of locomotor activity. However, only the primates treated with the combo demonstrated a significant improvement of motor disability and this effect was not accompanied by abnormal movements. These results confirm, in primates, what was previously observed with unilateral 6-OHDA-lesioned rats, namely that the combination treatment is able to restore good quality motor activity in the absence of motor complications and in the absence of stereotyped behaviour that would prevent normal exploration.

In preclinical models, rather than habituation, current dopaminergic therapies typically give rise to behavioural sensitization which translates to a progressive enhancement of motor activity by repeated administration of stimulant drugs when given in association with a specific testing context [31]. A phenomenon not only observed with psychostimulants (e.g. cocaine and amphetamine), but also attributed to treatment with L-Dopa in 6-OHDA-lesioned rodents [32] and MPTP-treated marmosets [33]. This L-Dopa-induced phenomenon is likely to be related to the development of LIDs. Even if the experiment did not address the chronic effect of the combination, the MPTP-treated marmosets which had been L-Dopa-primed, did not show any signs of abnormal movements after treatment with the combination. This observation strongly suggests that the NR2B/A_2A_ antagonist combination does not possess a pro-dyskinetic potential in primates, and our extensive previous studies have confirmed that this is also the case in rodents [28]. In the absence of any experimental evidence supporting a biological rationale, the interpretation of the behavioural observations remain hypothetical. One might speculate that the improvement of motor disability observed under the A_2A_/NR2B combination is due to a restoration of the balance of the dysregulated circuits of the basal ganglia. First, the behavioural improvement could result from the attenuation of the enhanced A_2A_ receptor tone which is a characteristic of the 6-OHDA-lesioned rats [34]. This A_2A_ antagonist effect on behaviour could be further enhanced by the inhibition of the NR2B receptor since these two receptors share a common intracellular signaling pathway [14]. Consequently, the efficacy of Tozadenant would be enhanced by the co-treatment with Radiprodil since mutual inhibition would result in a significant benefit. Also, it has been shown that, in the hippocampus, the A_2A_ receptor elicits the phosphorylation of the NR2B subunit (Tyr1472) that is required for the mGLUR5-mediated potentiation of the NMDA response [35]. If a comparable phenomenon also occurs in the striatum, one might expect that blocking the A_2A_ activity could prevent the Fyn-mediated tyrosine NR2B phosphorylation process involved in the development of L-Dopa sensitization [36] and of dyskinesia [37,38] when the direct D1 striatal pathway is stimulated.

In summary, this study reproduces, in a bilateral primate model of Parkinson’s disease, the motor improvements observed with the Radiprodil/Tozadenant combination in unilaterally 6-OHDA-lesioned rats. The strength of the accumulated preclinical data using these two models suggests that the combination of two clinical candidate drugs, Radiprodil and Tozadenant, could bring motor improvements while being unlikely to cause mechanism-based side effects. If these results can be further validated in a clinical study, it would make a strong case for considering the combined administration of an NR2B and A_2A_ antagonist for the symptomatic treatment of parkinsonian patients.

## Supporting information

S1 Table Latin square for combined twice daily administration of Tozadenant and Radiprodil. (DOCX)

S1 Fig Pharmacokinetic profile of Radiprodil and Tozadenant in MPTP-treated marmosets at the pharmacological active doses. Plasma monitoring in the pharmacology assay where 150 mg/kg Tozadenant and 2 mg/kg Radiprodil were delivered in combination (referred to as combo) as a suspension in 1% methylcellulose containing 0.1% antifoam, 0.1% Tween 80 0.1% and 20% hydroxypropyl-β-cyclodextrin to MPTP-treated animals. Results are expressed as the means ± SEM of 4 animals. The dotted lines indicate the targeted concentrations found to be active in the 6-OHDA-lesioned rat assay (i.e. 522 ng/mL Radiprodil and 6054 ng/mL Tozadenant).
(EPS)

S2 Fig Absence of pharmacokinetic interaction between Radiprodil and Tozadenant. A pilot pharmacokinetic study where 30 mg/kg Tozadenant and 2 mg/kg Radiprodil were delivered as a suspension in 1% methylcellulose containing 0.1% antifoam and 0.1% tween 80 0.1% to MPTP-treated marmosets. To test potential pharmacokinetic interactions, the drugs were administered either alone or in combination (referred to as combo). Results are expressed as means ± SEM of 3 to 6 animals (varying sample size as a result of Latin square design).
(EPS)

S1 File Supporting information. (DOCX)

S2 File Raw data for the locomotor activity counts. (PDF)

S3 File Raw data for the disability scores. (PDF)

S4 File Raw data for the dyskinesia scores. (PDF)

## References

[1] Connolly BS, Lang AE (2014) Pharmacological treatment of Parkinson disease: a review. JAMA 311: 1670–1683. doi: 10.1001/jama.2014.3654 24756517 10.1001/jama.2014.3654

[2] Hauser RA, Rascol O, Korczyn AD, Jon Stoessl A, Watts RL, et al (2007) Ten-year follow-up of Parkinson's disease patients randomized to initial therapy with ropinirole or levodopa. Mov Disord 22: 2409–2417. doi: 10.1002/mds.21743 17894339 10.1002/mds.21743

[3] Schiffmann SN, Fisone G, Moresco R, Cunha RA, Ferre S (2007) Adenosine A2A receptors and basal ganglia physiology. ProgNeurobiol 83: 277–292. 10.1016/j.pneurobio.2007.05.001 PMC2148496 17646043

[4] Fuxe K, Agnati LF, Jacobsen K, Hillion J, Canals M, et al (2003) Receptor heteromerization in adenosine A2A receptor signaling: relevance for striatal function and Parkinson's disease. Neurology 61: S19–S23. 14663004 10.1212/01.wnl.0000095206.44418.5c

[5] Bibbiani F, Oh JD, Petzer JP, Castagnoli N Jr, Chen JF, et al (2003) A2A antagonist prevents dopamine agonist-induced motor complications in animal models of Parkinson's disease. ExpNeurol 184: 285–294. 10.1016/s0014-4886(03)00250-4 14637099

[6] Pinna A, Fenu S, Morelli M (2001) Motor stimulant effects of the adenosine A(2A) receptor antagonist SCH 58261 do not develop tolerance after repeated treatments in 6-hydroxydopamine-lesioned rats. Synapse 39: 233–238. 10.1002/1098-2396(20010301)39:3<233::AID-SYN1004>3.0.CO;2-K 11169767

[7] Shook BC, Rassnick S, Osborne MC, Davis S, Westover L, et al (2010) In vivo characterization of a dual adenosine A2A/A1 receptor antagonist in animal models of Parkinson's disease. JMedChem 53: 8104–8115. 10.1021/jm100971t 20973483

[8] Kanda T, Jackson MJ, Smith LA, Pearce RK, Nakamura J, et al (1998) Adenosine A2A antagonist: a novel antiparkinsonian agent that does not provoke dyskinesia in parkinsonian monkeys. Ann Neurol 43: 507–513. doi: 10.1002/ana.410430415 9546333 10.1002/ana.410430415

[9] Hodgson RA, Bertorelli R, Varty GB, Lachowicz JE, Forlani A, et al (2009) Characterization of the potent and highly selective A2A receptor antagonists preladenant and SCH 412348 [7-[2-[4–2,4-difluorophenyl]-1-piperazinyl]ethyl]-2-(2-furanyl)-7H-pyrazol o[4,3-e][1, 2, 4]triazolo[1,5-c]pyrimidin-5-amine] in rodent models of movement disorders and depression. J Pharmacol Exp Ther 330: 294–303. doi: 10.1124/jpet.108.149617 19332567 10.1124/jpet.108.149617

[10] Hallett PJ, Standaert DG (2004) Rationale for and use of NMDA receptor antagonists in Parkinson's disease. Pharmacol Ther 102: 155–174. doi: 10.1016/j.pharmthera.2004.04.001 15163596 10.1016/j.pharmthera.2004.04.001

[11] Duty S (2012) Targeting glutamate receptors to tackle the pathogenesis, clinical symptoms and levodopa-induced dyskinesia associated with Parkinson's disease. CNSDrugs 26: 1017–1032. 10.1007/s40263-012-0016-z 23114872

[12] Loschmann PA, De Groote C, Smith L, Wullner U, Fischer G, et al (2004) Antiparkinsonian activity of Ro 25–6981, a NR2B subunit specific NMDA receptor antagonist, in animal models of Parkinson's disease. Exp Neurol 187: 86–93. doi: 10.1016/j.expneurol.2004.01.018 15081591 10.1016/j.expneurol.2004.01.018

[13] Nash JE, Fox SH, Henry B, Hill MP, Peggs D, et al (2000) Antiparkinsonian actions of ifenprodil in the MPTP-lesioned marmoset model of Parkinson's disease. Exp Neurol 165: 136–142. doi: 10.1006/exnr.2000.7444 10964492 10.1006/exnr.2000.7444

[14] Nash JE, Brotchie JM (2000) A common signaling pathway for striatal NMDA and adenosine A2a receptors: implications for the treatment of Parkinson's disease. J Neurosci 20: 7782–7789. 11027242 10.1523/JNEUROSCI.20-20-07782.2000 PMC6772864

[15] Michel A, Downey P, Nicolas JM, Scheller D (2014) Unprecedented Therapeutic Potential with a Combination of A2A/NR2B Receptor Antagonists as Observed in the 6-OHDA Lesioned Rat Model of Parkinson's Disease. PLoS One 9: e114086 doi: 10.1371/journal.pone.0114086 25513815 10.1371/journal.pone.0114086 PMC4267740

[16] Michel A, Downey P, Van Damme X, De Wolf C, Schwarting R, et al (2015) Behavioural Assessment of the A2a/NR2B Combination in the Unilateral 6-OHDA-Lesioned Rat Model: A New Method to Examine the Therapeutic Potential of Non-Dopaminergic Drugs. PLoS One 10: e0135949 doi: 10.1371/journal.pone.0135949 26322641 10.1371/journal.pone.0135949 PMC4555651

[17] Fernandez HH, Greeley DR, Zweig RM, Wojcieszek J, Mori A, et al (2010) Istradefylline as monotherapy for Parkinson disease: results of the 6002-US-051 trial. ParkinsonismRelat Disord 16: 16–20. 10.1016/j.parkreldis.2009.06.008 19616987

[18] Nutt JG, Gunzler SA, Kirchhoff T, Hogarth P, Weaver JL, et al (2008) Effects of a NR2B selective NMDA glutamate antagonist, CP-101,606, on dyskinesia and Parkinsonism. Mov Disord 23: 1860–1866. doi: 10.1002/mds.22169 18759356 10.1002/mds.22169 PMC3390310

[19] Addy C, Assaid C, Hreniuk D, Stroh M, Xu Y, et al (2009) Single-dose administration of MK-0657, an NR2B-selective NMDA antagonist, does not result in clinically meaningful improvement in motor function in patients with moderate Parkinson's disease. J Clin Pharmacol 49: 856–864. doi: 10.1177/0091270009336735 19491335 10.1177/0091270009336735

[20] Oertel WH (2017) Recent advances in treating Parkinson's disease. F1000Res 6: 260 doi: 10.12688/f1000research.10100.1 28357055 10.12688/f1000research.10100.1 PMC5357034

[21] Hauser RA, Olanow CW, Kieburtz KD, Pourcher E, Docu-Axelerad A, et al (2014) Tozadenant (SYN115) in patients with Parkinson's disease who have motor fluctuations on levodopa: a phase 2b, double-blind, randomised trial. Lancet Neurol 13: 767–776. doi: 10.1016/S1474-4422(14)70148-6 25008546 10.1016/S1474-4422(14)70148-6

[22] PBR SW (2010) Radiprodil Trial Fails To Show Reductions In Daily Pain Scores: Forest Labs, Gedeon Richter. In: News CT, editor. http://clinicaltrials.pharmaceutical-business-review.com/news/radiprodil_trial_fails_to_show_reductions_in_daily_pain_scores_forest_labs_gedeon_richter_100629.

[23] Jackson MJ, Jenner P (2012) The MPTP-treated primate, with specific reference to the use of the common marmoset (Callithrix jacchus) Animal models of movement disorders: *Neuromethods*. Lane Emma L.; Dunnett Stephen B. ed. pp. 371–395.

[24] Pearce RK, Jackson M, Smith L, Jenner P, Marsden CD (1995) Chronic L-DOPA administration induces dyskinesias in the 1-methyl-4- phenyl-1,2,3,6-tetrahydropyridine-treated common marmoset (Callithrix Jacchus). Mov Disord 10: 731–740. doi: 10.1002/mds.870100606 8749992 10.1002/mds.870100606

[25] Mancel V, Mathy FX, Boulanger P, English S, Croft M, et al (2016) Pharmacokinetics and metabolism of [14C]-tozadenant (SYN-115), a novel A2a receptor antagonist ligand, in healthy volunteers. Xenobiotica: 1–14. 10.1080/00498254.2016.1221164 27489076

[26] Kanda T, Tashiro T, Kuwana Y, Jenner P (1998) Adenosine A2A receptors modify motor function in MPTP-treated common marmosets. Neuroreport 9: 2857–2860. 9760134 10.1097/00001756-199808240-00032

[27] Grondin R, Bedard PJ, Hadj TA, Gregoire L, Mori A, et al (1999) Antiparkinsonian effect of a new selective adenosine A2A receptor antagonist in MPTP-treated monkeys. Neurology 52: 1673–1677. 10331698 10.1212/wnl.52.8.1673

[28] Michel A, Downey P, Montel F, Scheller D, Christophe B (2013) Methods for treating parkinson's disease: Google Patents.

[29] Bronstein PM, Neiman H, Wolkoff DF, Levine JM (1974) The development of habituation in the rat. Animal Learning & Behavior 2: 92–96.

[30] Ramaswami M (2014) Network plasticity in adaptive filtering and behavioral habituation. Neuron 82: 1216–1229. doi: 10.1016/j.neuron.2014.04.035 24945768 10.1016/j.neuron.2014.04.035

[31] Tirelli E, Michel A, Brabant C (2005) Cocaine-conditioned activity persists for a longer time than cocaine-sensitized activity in mice: implications for the theories using Pavlovian excitatory conditioning to explain the context-specificity of sensitization. Behav Brain Res 165: 18–25. doi: 10.1016/j.bbr.2005.06.029 16137776 10.1016/j.bbr.2005.06.029

[32] Carey RJ (1991) Chronic L-dopa treatment in the unilateral 6-OHDA rat: evidence for behavioral sensitization and biochemical tolerance. Brain Res 568: 205–214. 1814568 10.1016/0006-8993(91)91399-l

[33] Ando K, Inoue T, Itoh T (2014) L-DOPA-induced behavioral sensitization of motor activity in the MPTP-treated common marmoset as a Parkinson's disease model. Pharmacol Biochem Behav 127: 62–69. doi: 10.1016/j.pbb.2014.10.009 25449794 10.1016/j.pbb.2014.10.009

[34] Pinna A, Corsi C, Carta AR, Valentini V, Pedata F, et al (2002) Modification of adenosine extracellular levels and adenosine A(2A) receptor mRNA by dopamine denervation. EurJPharmacol 446: 75–82. 10.1016/s0014-2999(02)01818-6 12098587

[35] Sarantis K, Tsiamaki E, Kouvaros S, Papatheodoropoulos C, Angelatou F (2015) Adenosine A(2)A receptors permit mGluR5-evoked tyrosine phosphorylation of NR2B (Tyr1472) in rat hippocampus: a possible key mechanism in NMDA receptor modulation. J Neurochem 135: 714–726. doi: 10.1111/jnc.13291 26303340 10.1111/jnc.13291

[36] Dunah AW, Sirianni AC, Fienberg AA, Bastia E, Schwarzschild MA, et al (2004) Dopamine D1-dependent trafficking of striatal N-methyl-D-aspartate glutamate receptors requires Fyn protein tyrosine kinase but not DARPP-32. Mol Pharmacol 65: 121–129. doi: 10.1124/mol.65.1.121 14722243 10.1124/mol.65.1.121

[37] Ba M, Kong M, Ma G (2015) Postsynaptic density protein 95-regulated NR2B tyrosine phosphorylation and interactions of Fyn with NR2B in levodopa-induced dyskinesia rat models. Drug Des Devel Ther 9: 199–206. doi: 10.2147/DDDT.S75495 25565773 10.2147/DDDT.S75495 PMC4278739

[38] Kong M, Ba M, Liu C, Zhang Y, Zhang H, et al (2015) NR2B antagonist CP-101,606 inhibits NR2B phosphorylation at tyrosine-1472 and its interactions with Fyn in levodopa-induced dyskinesia rat model. Behav Brain Res 282: 46–53. doi: 10.1016/j.bbr.2014.12.059 25576965 10.1016/j.bbr.2014.12.059

