# Supplementary material for: Antiparkinsonian effects of the "Radiprodil and Tozadenant" combination in MPTP-treated marmosets
Source: PLoS One. 2017 Aug 30;12(8):e0182887. doi: 10.1371/journal.pone.0182887 (PMC5576667; doi:10.1371/journal.pone.0182887)
Supplement: S1 Table — (DOCX) [file pone.0182887.s001.docx]

| Animal I.D. | Subject no. | Day 1 | Day 2 | Day 3 | Day 4 |
| --- | --- | --- | --- | --- | --- |
| 9046 | 1 | Tozadenant 150 mg/kg | vehicle | Tozadenant 150 mg/kg + rad 2 | Radiprodil 2 mg/kg |
| 9057 | 5 | Tozadenant 150 mg/kg + rad 2 | vehicle | Radiprodil 2 mg/kg | Tozadenant 150 mg/kg |
| K12 | 7 | Radiprodil 2 mg/kg | Tozadenant 150 mg/kg + rad 2 | vehicle | Tozadenant 150 mg/kg |
| Y010 | 3 | Tozadenant 150 mg/kg | vehicle | Tozadenant 150 mg/kg + rad 2 | Radiprodil 2 mg/kg |
| 1a | 11 | vehicle | Radiprodil 2 mg/kg | Tozadenant 150 mg/kg | Tozadenant 150 mg/kg + rad 2 |
| X000 | 4 | Tozadenant 150 mg/kg + rad 2 | Tozadenant 150 mg/kg | Radiprodil 2 mg/kg | vehicle |
|  |  |  |  |  |  |
| px36 | 6 | toz 150 + rad 2 | Tozadenant 150 mg/kg | Radiprodil 2 mg/kg | vehicle |
| 9108 | 2 | Tozadenant 150 mg/kg | vehicle | Tozadenant 150 mg/kg + rad 2 | Radiprodil 2 mg/kg |
| PX17 | 8 | Radiprodil 2 mg/kg | Tozadenant 150 mg/kg + rad 2 | vehicle | Tozadenant 150 mg/kg |
| V215 | 9 | Radiprodil 2 mg/kg | Tozadenant 150 mg/kg + rad 2 | vehicle | Tozadenant 150 mg/kg |
| PX31 | 12 | vehicle | Radiprodil 2 mg/kg | Tozadenant 150 mg/kg | Tozadenant 150 mg/kg + rad 2 |
| KCL7 | 10 | vehicle | Radiprodil 2 mg/kg | Tozadenant 150 mg/kg | Tozadenant 150 mg/kg + rad 2 |
