# Supplementary material for: Antiparkinsonian effects of the "Radiprodil and Tozadenant" combination in MPTP-treated marmosets
Source: PLoS One. 2017 Aug 30;12(8):e0182887. doi: 10.1371/journal.pone.0182887 (PMC5576667; doi:10.1371/journal.pone.0182887)
Supplement: S1 File — (DOCX) [file pone.0182887.s004.docx]

**Supporting Information**

**Antiparkinsonian effects of the “Radiprodil and Tozadenant” combination in MPTP-treated marmosets.**

Anne Michel^1*^, Jean-Marie Nicolas^1^, Sarah Rose^3^, Michael Jackson^3^, Peter Colman^2^, Willy Briône^1^, David Sciberras^1^, Pierandrea Muglia^1^, Dieter K. Scheller^1^, Martin Citron^1^, Patrick Downey^1^.

^1^UCB BioPharma, Braine L’Alleud, Belgium.

^2^ UCB BioPharma, Slough, UK.

^3^ King’s College, Institute of Pharmaceutical Science, London, UK.

**Methods used to minimize any suffering for the primates.**

- Specialised post MPTP treatment after care was provided and included hand-feeding of fluids and liquidised high protein/energy food, bathing and cleaning and use of padded/insulated recovery units equipped with heated blankets.
- Social housing in male/female pairs (males vasectomised) in large cages in rooms with multiple cages  allowing visual and audio contact with other animals.
- A choice of sleeping areas were provided in each cage  including soft hammocks, swinging buckets and padded nest boxes.
- Extensive use of wooden ladders/cage furniture to assist mobility of bradykinetic animals
- Puzzle foraging/feeding utilising novel objects to provide stimulus.
- Animals were trained using positive reward  reinforcement to sit on balance for weighing and to enter carrying boxes for transportation.

**Behavioural analysis for the MPTP-treated Marmosets**

**Locomotor activity**

Basal locomotor activity was assessed during the 1 hour acclimatisation period. Each automated test unit (50 x 60 x 90 cm) was fitted with a clear perspex door to facilitate observation and equipped with 8 horizontally orientated photoelectric emitters/detectors (light beams), arranged so as to permit maximum assessment of perch and climbing activity. Interruption of a light beam was automatically recorded as a single locomotor count. Locomotor activity was assessed as the number of light beam interruptions accumulated in 10- and 30-minute intervals for 10 hours following drug treatment [[1](#_ENREF_1)].

**Motor Disability**

Motor disability was assessed simultaneously with locomotor activity through a one-way mirror by experienced observers blinded to treatment. Basal disability was assessed during the 1 hour acclimatisation period and once every 30 minutes after drug treatment for 10 hours using an established motor disability rating scale; alertness (normal = 0, reduced = 1, sleepy = 2); checking (present = 0, reduced = 1, absent = 2); posture (normal = 0, abnormal trunk +1, abnormal tail + 1, abnormal limbs + 1, flexed = 4); balance (normal = 0, impaired = 1, unstable = 2, spontaneous falls = 3); reaction to stimuli (normal = 0, reduced = 1, slow = 2, absent = 3); vocalisation (normal = 0, reduced = 1, absent = 2); motility (normal = 0, bradykinesia = 1, akinesia = 2). These values were summed, a maximum score of 18 indicating severe motor disability, a minimum score of 0 indicating maximum reversal of motor disability [[1](#_ENREF_1),[2](#_ENREF_2)]. A score of 8 is considered as the limit for indicating significant motor improvement.

**Dyskinesia**

Dyskinesia was assessed simultaneously with motor disability by experienced observers blinded to treatment. Basal dyskinesia was assessed during the 1 hour acclimatisation period and once every 30 minutes after drug treatment for 10 hours using an established dyskinesia rating scale ; 0 = absent; 1= mild, fleeting and rare dyskinetic postures and movements; 2 = moderate: more prominent abnormal movements, but not significantly affecting normal behaviour; 3 = marked, frequent and at times continuous dyskinesia affecting the normal pattern of activity; 4 = severe, virtually continuous dyskinetic activity, disabling to the animal and replacing normal behaviour.

**Pharmacokinetic method description.**

After internal standard addition, samples were treated with 8 volumes of acetonitrile. Supernatants were collected and analyzed on an API5000 mass spectrometer (ABSciex, Framingham, MA, USA) equipped with a turbo ion spray source and interfaced with an Agilent 1290 Infinity LC system (Agilent Technologies, Waldbroon, Germany). The mass spectrometer was operated in the positive ionization mode with Multiple Reactions Monitoring (m/z 407 to 292 for Tozadenant, 398 to 109 for Radiprodil). A Waters HSS T3 column (2.1×30 mm, 1.8 µm) was used. The mobile phase consisted of 100% water with 0.1% formic acid (phase A) and 100% acetonitrile with 0.1% formic acid (phase B). The mobile phase was delivered at a 1 mL/min flow rate operated in a linear gradient mode.

**Pharmacological status of the primates before testing.**

The MPTP lesion is not progressive and the period between MPTP-treatment and study should not alter the data. Animals were L-Dopa primed (8 mg/kg, po) for up to twenty-eight days within a few months of MPTP treatment until they expressed moderate to severe dyskinesia. They were then re-challenged immediately prior to any study as part of the animal selection to ensure that they were still primed.

**Drug regimen: Combined administration of Tozadenant plus Radiprodil twice daily.**

This experiment investigated the effects on motor deficits of Radiprodil plus Tozadenant twice daily at an interval of 5 hrs according to a modified latin square design in n=12 MPTP-treated marmosets. At the time of the second treatment a novel object (cotton reel, table tennis ball) was placed into the cage. Experiments were performed twice weekly with an intervening period of at least 2 days. Treatments: Tozadenant (150 mg/kg) plus Radiprodil (2.0 mg/kg), Tozadenant (150 mg/kg) plus vehicle, Radiprodil (2.0 mg/kg) plus vehicle, vehicle + vehicle.

1. Smith LA, Jackson MJ, Johnston L, Kuoppamaki M, Rose S, et al. (2006) Switching from levodopa to the long-acting dopamine D2/D3 agonist piribedil reduces the expression of dyskinesia while maintaining effective motor activity in MPTP-treated primates. Clin Neuropharmacol 29: 112-125.

2. Iravani MM, Costa S, Al Bargouthy G, Jackson MJ, Zeng BY, et al. (2005) Unilateral pallidotomy in 1-methyl-4-phenyl-1,2,3,6-tetrahydropyridine-treated common marmosets exhibiting levodopa-induced dyskinesia. Eur J Neurosci 22: 1305-1318.
