# Supplementary material for: Antiparkinsonian effects of the "Radiprodil and Tozadenant" combination in MPTP-treated marmosets
Source: PLoS One. 2017 Aug 30;12(8):e0182887. doi: 10.1371/journal.pone.0182887 (PMC5576667; doi:10.1371/journal.pone.0182887)
Supplement: S2 File — (PDF) [file pone.0182887.s005.pdf]

Locomotor activity

TREATMENT	SUBJECTS	30 MIN	60 MIN	90 MIN	120 MIN	150 MIN	180 MIN	210 MIN	240 MIN	270 MIN	300 MIN	330 MIN	360 MIN	390 MIN	420 MIN	450 MIN	480 MIN	510 MIN	540 MIN	570 MIN	600 MIN
**Vehicle**	9046	34	0	0	33	0	0	0	48	53	52	147	13	3	94	117	88	42	113	141	312
**Vehicle**	9057	155	24	19	38	6	3	5	7	7	22	52	5	10	26	74	67	102	95	155	128
**Vehicle**	9108	39	5	0	0	0	0	0	0	0	0	30	15	2	0	0	0	0	16	11	0
**Vehicle**	1A	7	1	154	177	72	0	33	0	0	0	15	0	0	0	0	0	0	1	215	112
**Vehicle**	KCL12	44	30	46	36	20	13	16	15	19	12	28	9	24	26	7	18	20	24	55	97
**Vehicle**	KCL7	489	75	84	95	63	25	73	21	54	43	123	24	17	49	24	134	153	175	186	253
**Vehicle**	PX 17	54	25	44	43	24	16	20	12	40	50	54	0	24	35	38	73	29	72	109	90
**Vehicle**	PX31	307	16	86	35	41	17	13	19	8	81	85	62	237	120	116	123	291	522	330	344
**Vehicle**	PX36	94	90	66	55	82	69	35	28	12	28	127	52	52	73	132	39	130	121	336	393
**Vehicle**	V215	39	0	29	0	6	13	39	56	45	111	50	31	107	73	150	123	85	142	107	151
**Vehicle**	X000	116	43	7	6	1	18	153	30	22	55	140	69	1	20	181	50	34	155	47	113
**Vehicle**	Y010	129	14	45	135	46	0	5	3	0	79	136	5	1	135	109	66	93	150	173	151
**Radiprodil 2,0**	9046	48	43	252	208	119	153	189	327	55	197	105	83	195	242	135	271	303	157	223	354
**Radiprodil 2,0**	9057	53	48	40	144	435	220	223	256	86	100	127	92	219	188	168	184	112	271	177	290
**Radiprodil 2,0**	9108	27	3	0	5	10	12	8	0	8	0	29	27	55	1	0	3	1	2	0	5
**Radiprodil 2,0**	1A	86	0	0	151	0	0	2	371	1050	863	389	0	0	0	456	231	831	939	829	1068
**Radiprodil 2,0**	KCL12	64	20	58	24	67	40	31	32	5	17	50	4	0	9	3	11	27	35	185	120
**Radiprodil 2,0**	KCL7	223	182	574	273	238	345	107	126	222	68	241	105	817	466	666	402	380	375	946	420
**Radiprodil 2,0**	PX17	113	76	167	85	68	85	23	0	66	9	111	27	12	46	16	76	133	176	124	163
**Radiprodil 2,0**	PX31	208	532	783	468	790	625	487	525	515	342	516	927	566	1118	1398	1677	3683	1898	2889	2129
**Radiprodil 2,0**	PX36	280	428	216	289	324	182	104	129	256	231	284	320	262	224	169	131	169	221	290	369
**Radiprodil 2,0**	V215	36	6	351	341	59	32	80	31	44	15	334	7	22	33	57	141	108	112	169	173
**Radiprodil 2,0**	X000	33	1	2	23	66	54	65	92	13	136	42	0	49	50	107	83	64	124	142	256
**Radiprodil 2,0**	Y010	133	39	18	46	93	71	26	42	65	25	120	20	70	116	126	92	100	151	67	195
**Tozadenant 150**	9046	36	32	63	49	118	34	170	226	38	140	245	110	163	192	113	174	137	91	198	236
**Tozadenant 150**	9057	26	10	0	0	0	20	29	23	15	16	72	111	143	119	177	24	363	278	89	477
**Tozadenant 150**	9108	28	0	0	1	0	0	0	0	0	0	25	0	0	0	0	0	1	7	1	0
**Tozadenant 150**	1A	9	0	0	0	542	0	204	70	0	0	14	0	0	0	0	0	0	0	325	548
**Tozadenant 150**	KCL12	33	13	20	19	29	16	29	22	14	17	66	69	41	34	46	23	36	70	74	126
**Tozadenant 150**	KCL7	228	196	111	122	52	28	87	17	82	92	141	44	134	182	76	251	183	530	317	344
**Tozadenant 150**	PX17	60	18	54	102	148	151	44	79	177	158	199	64	63	139	121	155	354	133	217	286
**Tozadenant 150**	PX31	315	182	596	228	497	95	246	49	260	157	623	185	184	121	365	217	303	384	240	393
**Tozadenant 150**	PX36	170	89	100	119	171	196	155	85	151	165	246	94	71	103	147	109	149	294	340	453
**Tozadenant 150**	V215	36	48	42	55	97	96	130	48	103	201	232	98	154	143	314	306	258	189	380	428
**Tozadenant 150**	X000	70	10	9	18	0	0	0	2	76	187	213	0	0	20	237	71	265	283	227	199
**Tozadenant 150**	YO10	64	31	104	160	72	68	125	122	95	89	127	110	48	42	10	54	121	62	114	13
**Toz 150 + Rad 2**	9046	30	106	161	233	396	214	202	285	250	186	378	275	542	299	429	528	470	489	512	943
**Toz 150 + Rad 2**	9057	45	95	515	628	425	343	575	303	279	263	254	418	519	782	586	397	584	850	856	643
**Toz 150 + Rad 2**	9108	33	74	154	250	76	11	25	0	3	8	26	14	60	81	130	107	127	7	7	16
**Toz 150 + Rad 2**	1A	73	0	0	0	0	187	706	493	354	490	1028	783	0	9	1451	614	1103	1453	745	1473
**Toz 150 + Rad 2**	KCL12	64	20	4	7	7	18	16	39	153	133	107	27	20	152	437	265	432	472	579	804
**Toz 150 + Rad 2**	KCL7	219	505	459	622	406	467	770	366	500	334	518	519	440	495	503	643	794	1239	1485	1493
**Toz 150 + Rad 2**	PX17	103	44	99	123	146	446	163	261	257	142	274	182	244	229	302	136	57	232	419	402
**Toz 150 + Rad 2**	PX31	312	259	808	676	526	718	712	569	406	389	384	635	1627	966	844	642	1075	1017	1704	1565
**Toz 150 + Rad 2**	PX36	240	615	776	283	387	398	260	224	269	283	518	520	557	437	251	527	608	605	726	768
**Toz 150 + Rad 2**	V215	28	142	215	324	97	115	136	215	597	334	630	586	922	1650	1690	1610	1720	1663	1665	1643
**Toz 150 + Rad 2**	X000	147	114	89	154	268	99	121	101	222	228	127	16	55	388	200	202	382	302	551	222
**Toz 150 + Rad 2**	Y010	111	71	142	163	163	161	198	166	204	210	171	112	233	140	228	241	211	328	281	303
