# Supplementary material for: Antiparkinsonian effects of the "Radiprodil and Tozadenant" combination in MPTP-treated marmosets
Source: PLoS One. 2017 Aug 30;12(8):e0182887. doi: 10.1371/journal.pone.0182887 (PMC5576667; doi:10.1371/journal.pone.0182887)
Supplement: S3 File — (PDF) [file pone.0182887.s006.pdf]

Disability scores

Treatment	Subjects	30 MIN	60 MIN	90 MIN	120 MIN	150 MIN	180 MIN	210 MIN	240 MIN	270 MIN	300 MIN	330 MIN	360 MIN	390 MIN	420 MIN	450 MIN	480 MIN	510 MIN	540 MIN	570 MIN	600 MIN
**vehicle**	9046	15	16	14	14	16	16	16	16	14	13	10	14	15	11	11	14	14	14	11	8
**vehicle**	9057	12	10	12	12	12	12	14	14	14	12	11	13	13	13	12	12	10	12	12	12
**vehicle**	9108	14	14	14	15	16	16	16	16	16	16	16	16	16	16	16	16	16	16	16	14
**vehicle**	1A	12	12	5	6	7	11	12	12	12	12	12	12	12	12	12	12	12	13	2	6
**vehicle**	KCL12	14	13	12	12	12	10	12	15	15	14	15	15	12	13	13	15	14	14	14	12
**vehicle**	KCL7	10	11	12	12	12	15	14	11	11	12	12	14	11	11	11	10	13	13	14	15
**vehicle**	PX17	14	14	14	14	14	12	14	14	14	13	14	14	14	14	14	14	14	14	14	14
**vehicle**	PX31	11	11	11	11	11	14	14	14	14	14	15	15	15	15	14	12	11	11	11	11
**vehicle**	PX36	10	10	11	10	10	10	11	11	12	12	11	12	12	12	12	12	12	11	10	10
**vehicle**	V215	14	13	13	13	13	13	11	10	11	10	9	5	9	5	14	8	8	10	11	11
**vehicle**	X000	7	8	8	10	10	10	12	12	12	12	7	12	12	12	12	12	12	10	10	10
**vehicle**	Y010	10	11	12	12	12	14	14	14	14	11	9	12	12	12	12	11	11	10	6	6
**Radiprodil 2,0**	9046	14	10	14	12	12	12	12	8	11	10	8	13	12	11	14	10	10	10	10	11
**Radiprodil 2,0**	9057	12	12	12	10	10	10	5	5	9	10	7	10	10	4	4	7	5	7	7	7
**Radiprodil 2,0**	9108	16	16	16	16	16	15	16	16	16	16	16	16	16	16	16	16	16	16	16	16
**Radiprodil 2,0**	1A	12	11	11	11	11	11	9	2	2	2	11	12	11	11	11	11	2	2	2	2
**Radiprodil 2,0**	KCL12	12	12	10	12	12	12	13	12	13	13	13	14	15	11	12	12	14	13	8	12
**Radiprodil 2,0**	KCL7	13	10	5	5	5	4	12	12	12	12	12	14	12	12	11	12	12	12	11	11
**Radiprodil 2,0**	PX17	11	11	11	11	11	12	12	11	11	11	11	10	12	12	12	14	7	11	11	11
**Radiprodil 2,0**	PX31	11	4	2	2	2	2	7	7	6	6	7	5	5	5	5	5	5	2	2	2
**Radiprodil 2,0**	PX36	8	7	8	8	7	10	9	9	10	10	10	10	10	10	10	12	12	11	11	11
**Radiprodil 2,0**	V215	12	13	10	10	10	13	13	6	7	7	7	11	11	1	11	4	12	12	10	11
**Radiprodil 2,0**	X000	11	11	11	11	10	11	8	11	11	9	10	10	9	7	7	11	11	10	5	5
**Radiprodil 2,0**	Y010	10	10	11	11	11	11	12	9	9	10	11	12	12	12	11	10	10	10	10	12
**Tozadenant 150**	9046	14	15	7	10	14	12	10	10	10	11	8	8	10	8	10	8	7	10	5	5
**Tozadenant 150**	9057	14	14	14	14	14	14	14	13	12	13	9	10	10	10	10	11	10	12	10	11
**Tozadenant 150**	9108	16	15	16	16	16	16	16	16	16	16	16	16	16	16	16	16	16	16	16	16
**Tozadenant 150**	1A	13	14	14	14	2	6	7	11	11	11	11	11	11	11	11	12	12	12	2	2
**Tozadenant 150**	KCL12	13	13	11	12	12	12	12	12	12	12	9	12	12	12	12	12	12	12	12	10
**Tozadenant 150**	KCL7	12	12	12	14	14	13	12	12	12	12	15	12	12	12	13	14	14	12	14	10
**Tozadenant 150**	PX17	14	12	10	11	10	10	12	12	12	12	12	12	12	12	11	12	11	11	11	12
**Tozadenant 150**	PX31	13	12	12	12	12	13	10	11	10	10	10	10	10	10	10	14	12	14	13	14
**Tozadenant 150**	PX36	5	6	6	6	6	6	8	8	8	8	6	8	8	7	7	8	7	7	7	9
**Tozadenant 150**	V215	14	11	5	5	5	5	4	4	4	5	7	5	5	5	5	9	9	11	4	12
**Tozadenant 150**	X000	5	11	7	10	11	11	11	11	11	5	10	12	12	12	12	12	3	5	5	5
**Tozadenant 150**	Y010	12	12	5	9	11	8	8	8	9	10	4	10	10	10	10	10	10	11	8	11
**Rad 2,0 + Toz 150**	9046	14	11	15	12	12	10	9	5	5	7	6	7	5	4	4	3	3	3	3	3
**Rad 2,0 + Toz 150**	9057	11	10	2	5	10	3	5	4	5	5	5	7	7	8	6	6	6	4	2	2
**Rad 2,0 + Toz 150**	9108	15	11	13	14	13	13	11	13	16	16	13	13	12	6	8	12	12	14	14	14
**Rad 2,0 + Toz 150**	1A	12	11	11	11	11	11	7	2	3	5	2	8	8	8	2	2	2	2	2	2
**Rad 2,0 + Toz 150**	KCL12	10	11	13	13	13	13	13	13	9	11	9	11	13	8	8	6	5	5	5	2
**Rad 2,0 + Toz 150**	KCL7	13	5	4	4	2	5	4	5	2	8	3	2	2	3	2	2	2	2	2	2
**Rad 2,0 + Toz 150**	PX17	12	12	11	11	11	11	11	10	10	11	12	10	12	11	11	11	11	11	8	5
**Rad 2,0 + Toz 150**	PX31	10	10	2	2	2	2	3	4	4	5	3	2	2	5	4	4	4	4	4	4
**Rad 2,0 + Toz 150**	PX36	9	9	5	5	6	4	3	4	4	4	4	6	5	5	10	4	3	3	3	3
**Rad 2,0 + Toz 150**	V215	11	3	2	2	3	2	9	9	5	9	2	6	5	2	2	2	2	2	2	2
**Rad 2,0 + Toz 150**	X000	11	7	5	5	6	4	8	8	9	6	7	11	12	12	11	10	9	3	2	3
**Rad 2,0 + Toz 150**	Y010	11	10	11	10	6	5	6	10	10	8	8	8	8	7	6	5	5	4	5	4
