# Supplementary material for: Antiparkinsonian effects of the "Radiprodil and Tozadenant" combination in MPTP-treated marmosets
Source: PLoS One. 2017 Aug 30;12(8):e0182887. doi: 10.1371/journal.pone.0182887 (PMC5576667; doi:10.1371/journal.pone.0182887)
Supplement: S4 File — (PDF) [file pone.0182887.s007.pdf]

Dyskinesia

Treatement	Animals	30 MIN	60 MIN	90 MIN	120 MIN	150 MIN	180 MIN	210 MIN	240 MIN	270 MIN	300 MIN	330 MIN	360 MIN	390 MIN	420 MIN	450 MIN	480 MIN	510 MIN	540 MIN	570 MIN	600 MIN
**vehicle**	9046	0	0	0	0	0	0	0	0	0	0	0	0	0	0	0	0	0	0	0	0
**vehicle**	9057	0	0	0	0	0	0	0	0	0	0	0	0	0	0	0	0	0	0	0	0
**vehicle**	9108	0	0	0	0	0	0	0	0	0	0	0	0	0	0	0	0	0	0	0	0
**vehicle**	1A	0	0	0	0	0	0	0	0	0	0	0	0	0	0	0	0	0	0	0	0
**vehicle**	KCL12	0	0	0	0	0	0	0	0	0	0	0	0	0	0	0	0	0	0	0	0
**vehicle**	KCL7	0	0	0	0	0	0	0	0	0	0	0	0	0	0	0	0	0	0	0	0
**vehicle**	PX17	0	0	0	0	0	0	0	0	0	0	0	0	0	0	0	0	0	0	0	0
**vehicle**	PX31	0	0	0	0	0	0	0	0	0	0	0	0	0	0	0	0	0	0	0	0
**vehicle**	PX36	0	0	0	0	0	0	0	0	0	0	0	0	0	0	0	0	0	0	0	0
**vehicle**	V215	0	0	0	0	0	0	0	0	0	0	0	0	0	0	0	0	0	0	0	0
**vehicle**	X000	0	0	0	0	0	0	0	0	0	0	0	0	0	0	0	0	0	0	0	0
**vehicle**	Y010	0	0	0	0	0	0	0	0	0	0	0	0	0	0	0	0	0	0	0	0
**Radiprodil 2,0**	9046	0	0	0	0	0	0	0	0	0	0	0	0	0	0	0	0	0	0	0	0
**Radiprodil 2,0**	9057	0	0	0	0	0	0	0	0	0	0	0	0	0	0	0	0	0	0	0	0
**Radiprodil 2,0**	9108	0	0	0	0	0	0	0	0	0	0	0	0	0	0	0	0	0	0	0	0
**Radiprodil 2,0**	1A	0	0	0	0	0	0	0	0	0	0	0	0	0	0	0	0	0	0	0	0
**Radiprodil 2,0**	KCL12	0	0	0	0	0	0	0	0	0	0	0	0	0	0	0	0	0	0	0	0
**Radiprodil 2,0**	KCL7	0	0	0	0	0	0	0	0	0	0	0	0	0	0	0	0	0	0	0	0
**Radiprodil 2,0**	PX17	0	0	0	0	0	0	0	0	0	0	0	0	0	0	0	0	0	0	0	0
**Radiprodil 2,0**	PX31	0	0	0	0	0	0	0	0	0	0	0	0	0	0	0	0	0	0	0	0
**Radiprodil 2,0**	PX36	0	0	0	0	0	0	0	0	0	0	0	0	0	0	0	0	0	0	0	0
**Radiprodil 2,0**	V215	0	0	0	0	0	0	0	0	0	0	0	0	0	0	0	0	0	0	0	0
**Radiprodil 2,0**	X000	0	0	0	0	0	0	0	0	0	0	0	0	0	0	0	0	0	0	0	0
**Radiprodil 2,0**	Y010	0	0	0	0	0	0	0	0	0	0	0	0	0	0	0	0	0	0	0	0
**Tozadenant 150**	9046	0	0	0	0	0	0	0	0	0	0	0	0	0	0	0	0	0	0	0	0
**Tozadenant 150**	9057	0	0	0	0	0	0	0	0	0	0	0	0	0	0	0	0	0	0	0	0
**Tozadenant 150**	9108	0	0	0	0	0	0	0	0	0	0	0	0	0	0	0	0	0	0	0	0
**Tozadenant 150**	1A	0	0	0	0	0	0	0	0	0	0	0	0	0	0	0	0	0	0	0	0
**Tozadenant 150**	KCL12	0	0	0	0	0	0	0	0	0	0	0	0	0	0	0	0	0	0	0	0
**Tozadenant 150**	KCL7	0	0	0	0	0	0	0	0	0	0	0	0	0	0	0	0	0	0	0	0
**Tozadenant 150**	PX17	0	0	0	0	0	0	0	0	0	0	0	0	0	0	0	0	0	0	0	0
**Tozadenant 150**	PX31	0	0	0	0	0	0	0	0	0	0	0	0	0	0	0	0	0	0	0	0
**Tozadenant 150**	PX36	0	0	0	0	0	0	0	0	0	0	0	0	0	0	0	0	0	0	0	0
**Tozadenant 150**	V215	0	0	0	0	0	0	0	0	0	0	0	0	0	0	0	0	0	0	0	0
**Tozadenant 150**	X000	0	0	0	0	0	0	0	0	0	0	0	0	0	0	0	0	0	0	0	0
**Tozadenant 150**	Y010	0	0	0	0	0	0	0	0	0	0	0	0	0	0	0	0	0	0	0	0
**Rad 2,0 + Tozadenant 150**	9046	0	0	0	0	0	0	0	0	0	0	0	0	0	0	0	0	0	0	0	0
**Rad 2,0 + Tozadenant 150**	9057	0	0	0	0	0	0	0	0	0	0	0	0	0	0	0	0	0	0	1	0
**Rad 2,0 + Tozadenant 150**	9108	0	0	0	0	0	0	0	0	0	0	0	0	0	0	0	0	0	0	0	0
**Rad 2,0 + Tozadenant 150**	1A	0	0	0	0	0	0	0	0	0	0	0	0	0	0	0	0	0	0	0	0
**Rad 2,0 + Tozadenant 150**	KCL12	0	0	0	0	0	0	0	0	0	0	0	0	0	0	0	0	0	0	0	2
**Rad 2,0 + Tozadenant 150**	KCL7	0	0	0	0	0	0	0	0	0	0	0	0	0	0	0	0	0	0	1	0
**Rad 2,0 + Tozadenant 150**	PX17	0	0	0	0	0	0	0	0	0	0	0	0	0	0	0	0	0	0	0	0
**Rad 2,0 + Tozadenant 150**	PX31	0	0	0	0	0	0	0	0	0	0	0	0	0	0	0	0	0	0	0	0
**Rad 2,0 + Tozadenant 150**	PX36	0	0	0	0	0	0	0	0	0	0	0	0	0	0	0	0	0	0	0	0
**Rad 2,0 + Tozadenant 150**	V215	0	0	0	0	0	0	0	0	0	0	0	0	0	0	0	0	0	0	0	0
**Rad 2,0 + Tozadenant 150**	X000	0	0	0	0	0	0	0	0	0	0	0	0	0	0	0	0	0	0	0	0
**Rad 2,0 + Tozadenant 150**	Y010	0	0	0	0	0	0	0	0	0	0	0	0	0	0	0	0	0	0	0	0
